# Facile and Reliable Thickness Identification of Atomically Thin Dichalcogenide Semiconductors Using Hyperspectral Microscopy

**DOI:** 10.3390/nano10030526

**Published:** 2020-03-14

**Authors:** Yu-Chung Chang, Yu-Kai Wang, Yen-Ting Chen, Der-Yuh Lin

**Affiliations:** 1 Department of Electrical Engineering, National Changhua University of Education, Changhua 500, Taiwan; tim7369836@gmail.com; 2 Department of Electronic Engineering, National Changhua University of Education, Changhua 500, Taiwan; h136111100k37@gmail.com

**Keywords:** 2D materials, transition metal dichalcogenides (TMDCs), hyperspectral microscopy, thickness identification, MoS_2_, MoSe_2_, WS_2_, WSe_2_, SnS_2_, SnSe_2_

## Abstract

Although large-scale synthesis of layered two-dimensional (2D) transition metal dichalcogenides (TMDCs) has been made possible, mechanical exfoliation of layered van der Waals crystal is still indispensable as every new material research starts with exfoliated flakes. However, it is often a tedious task to find the flakes with desired thickness and sizes. We propose a method to determine the thickness of few-layer flakes and facilitate the fast searching of flakes with a specific thickness. By using hyperspectral wild field microscopy to acquire differential reflectance and transmittance spectra, we demonstrate unambiguous recognition of typical TMDCs and their thicknesses based on their excitonic resonance features in a single step. Distinct from Raman spectroscopy or atomic force microscopy, our method is non-destructive to the sample. By knowing the contrast between different layers, we developed an algorithm to automatically search for flakes of desired thickness in situ. We extended this method to measure tin dichalcogenides, such as SnS_2_ and SnSe_2_, which are indirect bandgap semiconductors regardless of the thickness. We observed distinct spectroscopic behaviors as compared with typical TMDCs. Layer-dependent excitonic features were manifested. Our method is ideal for automatic non-destructive optical inspection in mass production in the semiconductor industry.

## 1. Introduction

Ever since the debut of graphene in 2004, layered two-dimensional (2D) materials have attracted tremendous attention due to their intriguing properties and great potential in various applications [1,2,3]. Their intrinsic feature of ultimate atomic thinness limit has triggered the development of transistors of atomic size [4,5]. Together with other advantages, 2D materials have demonstrated their great potential to push the frontier of semiconductor technology beyond Moore’s law [6,7,8,9]. However, the absence of an energy bandgap makes graphene less favorable for use in transistors. The field has quickly expanded into other layered 2D materials, predominantly semiconducting transition metal dichalcogenides (TMDCs), such as MoS_2_, WS_2_, MoSe_2_, and WSe_2_. These TMDCs have bandgaps in the visible wavelength range. In addition, many of the TMDCs exhibit thickness dependent bandgaps and electronic band structures. For example, the most studied TMDCs, MoS_2_ and WS_2_, are indirect materials in their bulk form but have direct bandgaps as monolayer crystals [5,10,11]. These 2D-TMDCs have thus emerged as an exciting class of atomically thin semiconductors for a new generation of electronic and optoelectronic devices. 

Despite the appealing properties of layered 2D materials, practical applications are largely hampered by the limited capability in producing controllable high quality uniform crystalline thin film in a large area. Although wafer-scale monolayer MoS_2_ synthesis has been reported [12,13,14], the growth mechanism is not fully understood [15,16]. The growth recipes are created by numerous empirical attempts due to the uncontrollability, which renders a slow process that cannot meet the high demand. Among the methods, mechanical exfoliation of bulk crystals remains the most efficient way to obtain high quality crystalline nanoflakes. The weak, van der Waals interactions between the layers of the corresponding bulk materials enable atomically thin layers of TMDCs to be isolated easily by mechanical exfoliation. In fact, every new layered 2D material research starts with mechanically exfoliated flakes [17,18]. However, due to the physical nature of the method, the exfoliation results in randomly distributed flakes of various thicknesses in small sizes. To identify the thickness of the flakes, typical methods are atomic force microscopy (AFM) [19], Raman spectroscopy [20,21], and optical microscopy (OM) [22,23]. AFM is the most adopted method to measure the thickness of 2D materials, but it takes a long time to scan only a small area. Besides, the scanning might cause damage to the sample surface. Raman spectroscopy requires a relatively high laser intensity, which might cause irreversible damage to the sample. In addition, for graphene and MoS_2_, the Raman spectra do not show a significant difference for crystal thickness of more than three layers. For some materials, the Raman signals do not show significant change even down to a monolayer. This makes Raman spectroscopy insufficient for accurate layer number identification [23]. On the contrary, OM is an easy-accessible, efficient, and nondestructive technique that enables rapid characterization of layered 2D materials. Nowadays, due to the layer-dependent optical contrast between the atomically thin 2D crystals and the substrate, optical microscopic techniques have been widely employed to identify the flakes and to determine their thickness [24,25].

Because the optoelectronic properties of few-layer TMDCs are highly layer-dependent, it is imperative to determine the thickness of the nanoflakes in an efficient and reliable manner. Although the contrast is layer-dependent due to the strong light-matter interaction of these 2D crystals, the contrast is easily influenced by illumination, sample alignment, and contaminations. Thus, this method only gives us a relative relation between different thicknesses, which is somewhat ambiguous. The flake thickness still needs to be verified by AFM. This renders an inefficient process that does not meet the requirement of the semiconductor industry. On the other hand, because the exfoliated flakes are often only a few micrometers in size, high magnification is needed to find the flakes, but this inevitably translates to a small field of view. It is very time-consuming and tedious to search the flakes manually. Besides, it is also difficult for human eyes to determine the flake thickness precisely. To this end, using machine learning to automatically search for flakes with the desired size and thickness has been demonstrated recently [25,26,27]. The artificial-intelligence (AI)-based techniques have enabled high-throughput autonomous assembly of van der Waals superlattices and heterostructures of 2D atomic crystals [28,29]. However, a large training data set (images) is needed for the machine learning algorithm to have a practical accuracy rate. For the intensively studied materials, such as graphene or MoS_2_, this might not be a big issue [28]. However, for novel 2D materials, it might be difficult to obtain so many few-layer flakes at the infant stage of research. In addition, machine learning-based technique can only identify a single material because the identification mechanism is based on color or optical contrast. It is desirable to identify multiple 2D materials simultaneously for the constructing of van der Waals heterostructures [30], the building block of functional devices.

Recently, reflectance and transmittance spectroscopy has been proven to be effective for the identification of few-layer TMDC thickness [24,31,32,33]. In this work, we adapted this technique in wild field hyperspectral microscopy to facilitate fast identification of flakes of desired thickness in a reliable and nondestructive manner. We demonstrate unambiguous recognition of typical TMDCs (MoS_2_, WS_2_, MoSe_2_, and WSe_2_) and their thicknesses based on their excitonic resonance features. Based on the spectroscopic features, we can identify multiple materials simultaneously. To facilitate the fast searching of flakes of appropriate sizes, we developed a microscope system that is able to look for flakes over a centimeter/millimeter-sized area. Our system is capable of automatic searching for flakes with the desired thickness. The reliability of our method is verified by the excellent agreement between the measured spectra and the theoretically calculated spectra based on Fresnel’s equations. We extend this method to measure tin dichalcogenides, such as SnS_2_ and SnSe_2_, which are indirect bandgap semiconductors regardless of the thickness. We observed distinct spectroscopic behaviors as compared with typical TMDCs. Layer-dependent spectroscopic features are evidently manifested. To our knowledge, this is the first spectroscopic study on the layer-dependent exciton evolution in few-layer tin dichalcogenides. This technique is ideal for high-throughput non-destructive optical identification of multiple kinds of 2D materials with the desired thickness for fundamental research or practical applications.

## 2. Materials and Methods 

All the crystals in this study were grown by a chemical vapor transport (CVT) method in our lab using the protocols described previously in [34,35,36,37]. Briefly, a horizontal three-zone furnace was utilized where the high (low) temperature zone was used as the reaction (growth) zone. Before the crystal growth, a quartz tube containing the transport agent and the crystal elements required was evacuated to below 10^−6^ Torr and securely sealed. The CVT process was initiated with a temperature gradient varying from 1000 °C in the reaction zone to 900 °C in the growth zone over a distance of about 30 cm, and the system was held in this condition over 500 h to provide sufficient diffusion and equilibration for the single-crystal synthesis. The CVT growth yielded large, thin, and shiny dichalcogenides crystals that were typically a few millimeters in lateral size with high crystallinity. The quality of the crystals and ratios of elements were verified by X-ray diffractometer (XRD), transmission electron microscopy (TEM), energy-dispersive spectroscopy (EDS), and Raman spectroscopy, as reported previously [34,35,37,38,39]. The few-layer nanosheets were prepared by mechanical exfoliation with UltraTape (1114WH100, Cleanroomtape Inc., Wilsonville, OR, USA). All thicknesses in this study were verified by atomic force microscopy (AFM).

**Figure 1:**
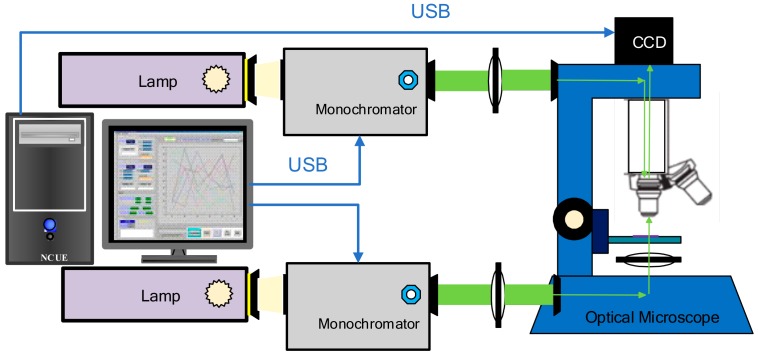
The schematic of the hyperspectral microscopic system. The excitation light sources are halogen lamps. The excitation light passes through a monochromator before entering the objective for both reflection mode and transmission mode. A CCD camera is used to capture images. A computer is used to synchronously control the monochromators and the camera for hyperspectral imaging.

In order to facilitate fast few-layer flake identification and thickness determination, we developed a wide-field hyperspectral microscopic system. The system is schematically shown in Figure 1. Our system is capable of measuring both reflection and transmission images. Our system is similar to what was demonstrated by Castellanos-Gomez et al. [31,32]. However, the spectra are calculated by integrating the intensities in a selected area instead of only one pixel. Therefore, a better signal-to-noise ratio (SNR) is achieved. The system was modified using a conventional microscope (BH2-UMA, Olympus) that is commonly used for 2D material inspection. The light sources are halogen lamps. The excitation light passes through a monochromator (CM110, Spectral Products Inc., Putnam, CT, USA) before entering the objective, respectively, for reflection mode and transmission mode. The monochromator allows one to select the excitation wavelength with a resolution below 1 nm. The spectrally resolved images were typically acquired in every 1 nm. A low noise coupled charge device (CCD) camera (Infinity3, Lumenera Inc., Ottawa, Ontario, Canada) was attached to the trinocular port of the microscope to capture images at different wavelengths. This system is capable of taking hyperspectral images for photons with energy between 1.4 eV to 3.1 eV (400 nm to 900 nm). The reflectance contrast and transmittance spectra are obtained by integrating the signals in the selected area of the image for the entire spectrum. The reflectance contrast is calculated according to Equation (1), where *I_sample_* is the reflection intensity from the sample, and *I_substrate_* is reflection from the 1 mm-thick glass slide. The transmittance *T* is calculated by dividing the transmission of the sample *T_sample_* with the background transmission *T*_0_, as *T* = *T_sample_*/*T*_0_.

(1) $$Reflectance\;Contrast=\frac{I_{sample}-I_{substrate}}{I_{sample}+I_{substrate}}$$

**Figure 2:**
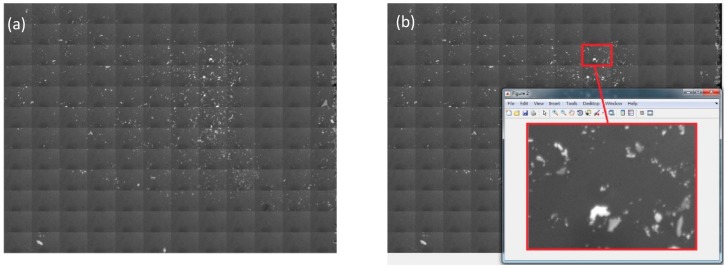
Illustration of the rapid sample finding microscope. (**a**) A wide field microscopic image combined with 12 × 12 small high resolution images. The total area is about 3 × 4 mm^2^. (**b**) When one clicks on a small area in the large image, a new window pops up with a magnified view of that area. The size of each small area is about 300 × 250 μm^2^. The magnification is 1600×.

**Figure 3:**
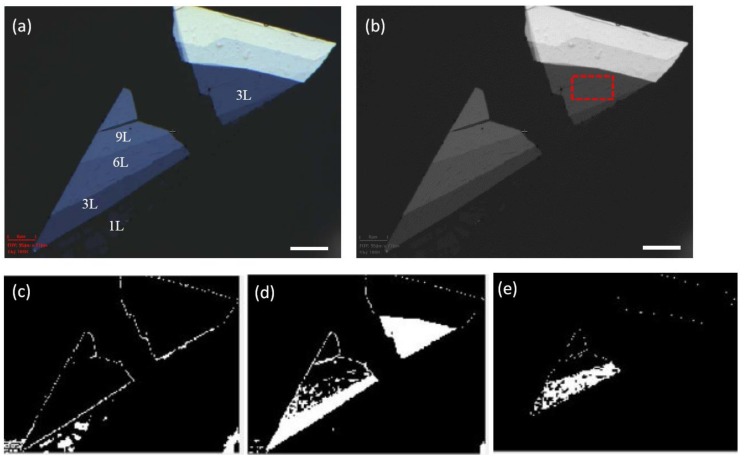
(**a**) Reflective optical microscope image of a MoS_2_ flake taken with a 100× objective. (**b**) Gray scale image after binarization. The red dashed rectangular indicates the selected area for spectral calculation. (**c**) The monolayer areas are highlighted in the image as the white areas by setting the threshold as 15–20. The boundaries of the flakes are also indicated. (**d**) The tri-layer areas are identified in the image as white areas by setting the threshold as 24–28. (**e**) The 6-layer areas are identified in the image as white areas. The white scale bar in the figure is 10 μm.

To facilitate fast searching of flakes with the desired thickness, we developed a semi-automatic technique using the hyperspectral imaging system. We used computer-controlled stepping-motors and the microscopic system to automatically take high-resolution images of the sample and put them together, as shown in Figure 2a. The large image consists of 144 small microscope pictures with 12 columns × 12 rows. The area of each small image is 250 × 300 μm^2^. The total viewable area is about 3 × 4 mm^2^. The magnification of each small image is 1600 times on the screen when using a 50× objective. Therefore, it is easier for human to identify the location of flakes of optimal sizes over a large area. If we want to have a closer look at a specific area, we just simply click on the small block and the high-resolution image opens in another window, as shown in Figure 2b. Once a few-layer flake is located, we can select an area on it to calculate the spectra, as shown in Figure 3. As will be discussed in the following section, the layer number can be determined unambiguously from the spectra. Different materials can also be distinguished by distinct spectroscopic features. We can then search for flakes of the desired thickness over the entire imaged area by the relative contrast between different layers. The relative contrast of the same material is layer-dependent [23], which has to be determined empirically. By setting a specific threshold, the flakes of the desired thickness can be identified automatically. 

The procedure for automatic identification of few-layer flakes of desired thickness is illustrated as follows: A high magnification image of a MoS_2_ flake is shown in Figure 3a. The layer number of each part is labeled on the flakes. The image is first converted to gray scale as shown in Figure 3b for edge detection. The 8-bit intensity scale is transformed to a 0–100 scale for binarization. First, we select an area to calculate the spectra as the red rectangular shown in the figure. If the selected area is determined to be a 3-layer MoS_2_ after comparing its spectra to our database and its intensity scale is 24–28, by setting the upper and lower binarization thresholds as 24 and 28, all tri-layer areas are identified in the image, as shown in Figure 3d. From the empirically obtained contrast between mono-layer and tri-layer, we can set the upper and lower thresholds for the mono-layer as 15 and 20, respectively. Similarly, all mono-layer flakes can be located in the images automatically, as shown in Figure 3c. By setting the thresholds as 34 and 36, the 6-layer area is identified, as shown in Figure 3e. Therefore, by knowing the thickness of a specific area, we can search for areas of the desired thickness. After the areas are identified, we can calculate the spectra of that area to confirm the thickness. 

In order to make our flake searching and layer-number identification procedure clearer, we summarize our method as the following steps:

Step 1. The machine automatically takes high-resolution microscopic images of the sample over a millimeter to centimeter size area. The human can identify the possible few-layer flakes over the large area.
Step 2. After a few-layer flake is located, we can select an area on it to calculate the spectra. By comparing the calculated spectra to the spectra in the database, we can identify the material species and determine its thickness.
Step 3. Using the layer-dependent relative contrast from the database, we can set specific thresholds for the automatic searching of flakes of the desired thickness over the entire imaged area.
Step 4. The human can look for flakes of appropriate size to calculate and compare its spectra to verify the thickness.

## 3. Results and Discussions

It is time-consuming and tedious to find mechanically exfoliated flakes of optimal size for further device manufacturing. In order to facilitate fast flakes identification, we have developed a semi-automatic flake searching method to identify flakes with the desired thickness, as described in the previous section. Because the exfoliated flakes are small and randomly distributed, high magnification is needed to find the flakes, which renders a small field of view. It takes a lot of time to search the flakes manually. Besides, it is also difficult for human eyes to determine the flake thickness precisely. To this end, using machine learning or AI-based techniques to automatically search for flakes with the desired size and thickness has been developed recently, as described in the introduction [25,26,27]. However, the AI-based techniques are not smart enough to explore thousands of possible novel layered 2D materials for future electronics. They can only be applied to relatively mature materials. Human intervention is necessary for the exploration of the unknown 2D territory. 

After finding a flake of few layers, a critical step before the further process is to identify its thickness. The optical contrast of few-layer 2D TMDCs is layer-dependent, so it can be used as a convenient parameter for thickness identification. However, using only optical contrast to distinguish the thickness is not accurate, as the reflectance contrast is not a monotonic function of the layer numbers, and it is easily influenced by non-uniform illumination, contaminations, sample-to-sample variations, etc. [17]. Each sample area has to be verified with AFM before layer identification can proceed [23]. Therefore, it is impossible to conduct a search over a large area. The searching process is interrupted by frequent AFM scans, which is inefficient and non-reliable.

Recently, it has been demonstrated that reflectance contrast spectra can be used to unambiguously determine the thickness of TMDC nanoflakes [24,40]. It is proposed that the spectroscopic technique can be employed as an alternative method to unambiguously determine the number of layers [24]. Here, we demonstrate using reflectance and transmittance spectra to unambiguously determine the layer numbers and use the information to facilitate fast and reliable identification of flakes with the desired thickness. The thickness determination and flake searching process can be completed in 5 min without having an AFM scan of the sample. 

**Figure 4:**
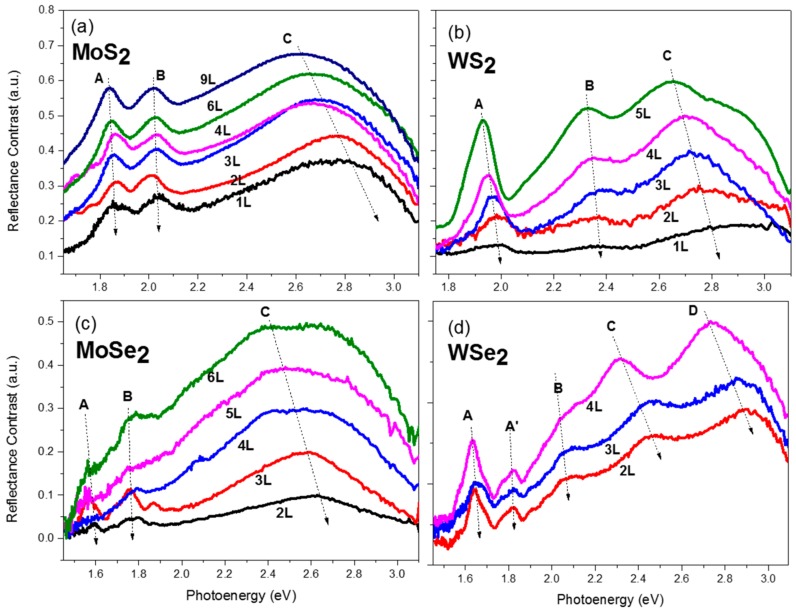
Reflectance contrast spectra of few-layer (**a**) MoS_2_, (**b**) WS_2_, (**c**) MoSe_2_, and (**d**) WSe_2_ crystals. The excitonic features are marked in the figures according to conventional notations. The thickness is labeled in the figures as monolayer (1L), bi-layer (2L), tri-layer (3L), and so on. The spectra are vertically shifted for visual clarity. The dotted arrow lines are guides for the eyes.

**Figure 5:**
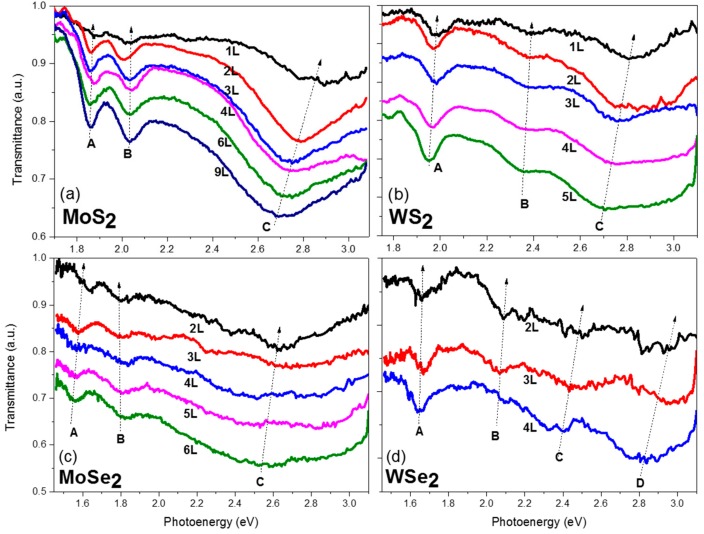
Transmission spectra of the same few-layer (**a**) MoS_2_, (**b**) WS_2_, (**c**) MoSe_2_, and (**d**) WSe_2_ flakes as in Figure 4. The spectra are vertically shifted for visual clarity. The dotted arrow lines are guides for the eyes.

Figure 4 shows reflectance contrast spectra of few-layer MoS_2_, WS_2_, MoSe_2_, and WSe_2_ of various thickness in a spectral range from 1.5 eV to 3.1 eV (400 nm to 850 nm). The transmittance spectra of the same respective flakes are shown in Figure 5. As is evident from the figures, the spectra show strong layer-dependent behavior. The excitonic peaks are labeled as A, B, and C (and D for WSe_2_) in the figures according to common nomenclature of semiconducting TMDCs in the literature [24,31,41]. The A exciton is the most studied one, which originated from the absorption transitions at the K point of the Brillouin zone from the upper valance band to the lowest conduction band. This peak exhibits a blue shift as the layer number decreases from few-layer to monolayer for the four TMDCs. It corresponds to the dominant peak in photoluminescence (PL) spectra. The peak shifts have also been seen in photoluminescence studies [41]. The B excitons occurring at slightly higher energy originate from the transitions from the split lower valance band to the conduction band. The position of the B peaks does not change as significantly because although the bandgap of the TMDCs increases as the thickness decreases, the spin-orbital coupling induced valance band split decreases, which compensates the energy difference [42]. 

Other than the A and B exciton peaks, a broader spectroscopic feature is prominent at the higher energy range from 2.3 to 3.1 eV, which is commonly referred to as the *C* (and *D* in WSe_2_) exciton peak. The strong light–matter interaction has been attributed to the transitions of multiple nearly degenerate states between the *Г* and *K* points of the Brillouin zone, where the conduction and valance bands are parallel to each other [24,43,44,45]. The electron–phonon interaction further contributed to the broadened resonance. As is noted by Castellanos-Gomez et al., this C excitonic feature is rarely mentioned in previous TMDCs studies, probably because most previous excitonic studies were conducted with PL using an excitation laser of a wavelength of about 500 nm (2.5 eV) [24,32]. 

As clearly seen in the figures, the *C* exciton peak exhibits a significant blue shift as the layer number decreases for the four TMDCs. The layer number can be determined solely by the peak position of the *C* peak. With both information in the reflectance and transmittance spectra, the layer number can be unambiguously determined. It is worth mentioning that when carefully investigating the reflectance contrast spectra of MoS_2_ (Figure 4a), the spectra of 3-layer (3L) and 4-layer (4L) cross each other. However, the photo-energy of the *C* peak of 3L is large than that of 4L. The layer number can thus be distinguished. In addition, because the transmission spectra were acquired at the same time, the differences in the 3L and 4L can be seen in the transmission spectra as well. The reason for the difference in the 3L and 4L spectra is because they are from flakes on different glass slides. For the spectra shown in Figure 4a, the mono-layer, 3-layer, 6-layer, and 9-layer are from the same flake, as shown in Figure 3a. The bi-layer, 4-layer samples are from another slide. The illumination, substrate, and ambient conditions might be different among samples. The tails of the spectra are easily influenced as the excitation light is weak at those ranges. It should be noted that even despite the different conditions of the sample, the layer-number can still be distinguished by spectroscopic features. This proves the superior capability of this technique for layer determination. 

Similarly, for other materials, most of the flakes of different layers are from different areas or slides. It is very difficult to find flakes of the desired thickness with appropriate sizes in the same area. For MoSe_2_ and WSe_2_, almost every curve in the figures is from a different flake. Probably because of the stronger interlayer interaction between the adjacent layers for the diselenides, it is more difficult to get few-layer flakes. Therefore, even though the flake-to-flake variation is large, we can still distinguish the thickness based on the peak positions of the C exciton. The A and B exciton peaks can be used to determine the material species.

**Figure 6:**
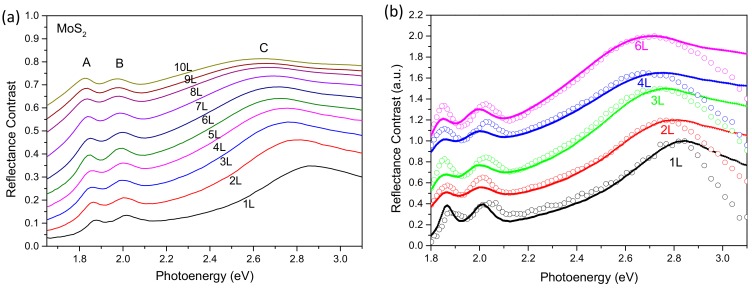
(**a**) Simulated reflectance contrast spectra of MoS_2_ using Fresnel’s equations from monolayer to 10-layer. (**b**) Overlay of the measured spectra with the simulation. The open circles are the measured data. The solid lines are the simulations. The curves are shifted in the vertical axis for visual clarity.

We used the complex refractive index of few-layer MoS_2_ provided by Yu et al. in [46] to calculate the reflectance contrast and transmission using Fresnel’s equations. The complex refractive indexes are measured by ellipsometry on CVD-grown films of specific thickness. The calculated reflectance contrast spectra of mono-layer to 10-layer MoS_2_ are shown in Figure 6a. The excitonic features are clearly identified in the spectra. Although the relative contrast between the A, B peaks and the C peak gets smaller as the layer number increases, the shift of the C exciton peak is monotonic and quite significant. We plotted the measured spectra with the theoretically calculated spectra together in Figure 6b. As seen in the figure, the peak positions of the C exciton perfectly match mutually. The quick drop of the measured spectra at the higher photoenergy tail (>2.8 eV) is due to weaker excitation light at the higher energy and the lower responsivity of the silicon CCD. This verification not only proved the reliability of our method, but also provided a convenient route for thickness determination. We can compare the measured spectrum to the calculated ones to determine the thickness if we know the complex reflective index or dielectric function of the material of different thickness. Once the thickness is determined, we can use the method described in the Materials and Methods to look for flakes with the desired thickness over the imaged area.

One major advantage of using spectroscopic features to determine the thickness of layered materials is that it can be applied to indirect bandgap semiconductor or non-semiconducting materials that have weak or no photoluminescence [32]. Tin disulfide (SnS_2_) and tin diselenide (SnSe_2_) have attracted much attention recently. They are layered 2D semiconductors with a group IV Sn atom replacing the transition metal in TMDCs. Their bandgaps do not have indirect-to-direct transitions as in the TMDCs [47,48,49]. The earth-abundant, low-toxicity, chemically and environmentally stable, and relatively easier availability characteristics have made them promising candidates for the widespread use of layered 2D semiconductors. During the last few years, few-layer and thin-film SnS_2_ and SnSe_2_ have been applied in several applications, such as field effect transistors with high on/off ratios [50,51], photocatalysis for high efficiency water-splitting using visible-light [52], gas sensing with high specificity [53], and photovoltaic devices with exceptional conversion efficiency [54]. The bandgap of SnS_2_ is in the range of 2.2–2.4 eV, and the bandgap of SnSe_2_ is in the range of 1–2 eV. Their alloys cover almost the entire range of the visible and infrared spectra [37], which makes them suitable materials for optoelectronic and light-harvesting applications [55,56]. However, a systematic study of few-layer tin dichalcogenides is still lacking. 

**Figure 7:**
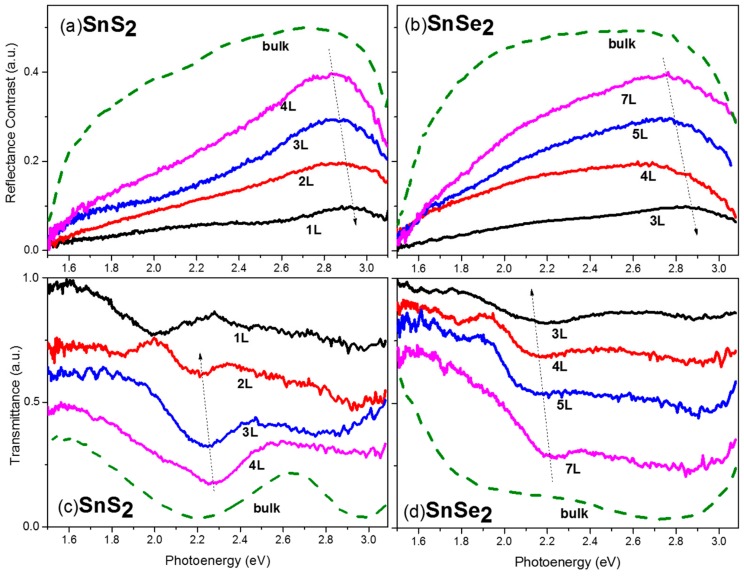
Reflectance contrast spectra of few-layer and bulk (**a**) SnS_2_ and (**b**) SnSe_2_. Transmission spectra of few-layer and bulk (**c**) SnS_2_ and (**d**) SnSe_2_. Some curves are shifted in the vertical axis for visual clarity. The thickness is labeled in the figures. The dotted arrow lines are guides for the eyes.

The reflectance contrast and transmission spectra of SnS_2_ and SnSe_2_ are shown in Figure 7. Prominent layer-dependent characteristics are revealed in the spectra. The spectra of bulk materials are presented for reference. The bulk spectra are measure from flakes of thicknesses 123.6 and 64.9 nm for SnS_2_ and SnSe_2_, respectively. Because the bandgaps of bulk SnS_2_ and SnSe_2_ are 2.3 eV and 1.2 eV, respectively, their reflectance contrast spectra are quite different. An absorptive feature is seen in the spectra of bulk SnS_2_ at about 2.2 eV, while the response of SnSe_2_ is almost flat over the entire measurement range because its bandgap is not in this range. This agrees with our intuitive expectation. An increase of transmission at about 2.6 eV for bulk SnS_2_ agrees with the measurement by Rusu et al. [57], where a drop of absorption is observed. 

Unlike the four TMDCs discussed previously, except for the broad peak at about 2.8–2.9 eV, there is no obvious excitonic feature in the reflectance spectra of few-layer SnS_2_ and SnSe_2_. The origin of the broad peak is due to large absorption at the ultraviolet range (above 3 eV) of the tin dichalcogenides. The peak is just the onset of an even broader peak, but not the real maximum. The broader peak is truncated only because the detector response is weaker at the higher energy tail. An obvious blue shift of the peaks is also observed as the layer number decreases, although not as large as the C peaks in the TMDCs. The shift should be due to a similar reason, namely an increase of the bandgap as the layer number shrinks, as reported in the theoretical calculation by Oleynik et al. [48]. According to the measurements by Rusu et al. [57], the reflective index of SnS_2_ is featureless in the visible to IR range at room temperature, which might account for the observed featureless reflectance contrast.

On the other hand, unlike the featureless reflectance contrast spectra, prominent layer-dependent excitonic feature evolution is present in the transmission spectra. The evolution of maximum peaks in the reflectance contrast spectra (*R*_max_), local minimums in transmittance spectra where the excitonic feature is present (*T*_min_), and the reported exciton binding energies (*Ex*) are summarized in Table 1. In contrast to the TMDCs, the excitonic features in the transmission spectra exhibit a red shift as the layer number decreases. We speculate the distinct behavior is due to the extraordinary large binding energy of SnS_2_ and SnSe_2_. Based on the ab initio density-function theory calculation of Sutter et al. [47], the bandgap of SnS_2_ is quite insensitive to the thickness for few-layer flakes, which is quite distinct to the TMDCs. This distinct behavior can be explained by the orbital composition of the conduction and valence band edges. In both bulk and monolayer SnS_2_, the valence band edge along *Г*-*M* is dominated by sulfur *P_x_* and *P_y_* (in-plane) orbitals. The conduction band edge at the *M* valley is a hybrid of half tin *s*-orbital and almost half sulfur *P_x_* and *P_y_* orbitals, while the electronic bands of MoS_2_ are dominated by the molybdenum d- and sulfur *p*-orbitals that are sensitive to interlayer coupling and confinement effects. The distinct configurations make the bandgap of SnS_2_ insensitive to both interlayer coupling and confinement.

Because the bandgap of SnS_2_ and SnSe_2_ is quite insensitive to the thickness for few-layer flakes, the shifting in the excitonic feature is mainly due to the change in the exciton binding energy. The excitonic binding energy *Ex* increases as the layer number decreases, which causes the red shifting of the excitonic absorption peak. For nanosheets thicker than 2-layer, the values of *T*_min_ are almost in arithmetic progression for both materials. The successive decrease of *T*_min_ is in accordance with the increase of *Ex* as the layer number shrinks. In the calculation of Oleynik et al., the exciton binding energy of SnS_2_ increases drastically from 0.3 eV to 0.9 eV when the thickness reduces from 2-layer to monolayer. The excitonic binding energy of mono-layer SnS_2_ is greater by a factor of two compared to typical TMDCs despite the comparable values in the bulk structures [48]. We also notice a drastic change in the location of the excitonic feature in the transmission spectra of SnS_2_ when thickness reduces from 2L to a monolayer. This is a strong evidence reflecting the above speculation. 

There are very limited spectroscopic studies about few-layer SnSe_2_ available in the literature. The bandgap for few-layer SnSe_2_ is above 2 eV, as reported by Nanda et al. [58], which is also evident in our transmission spectra. We start to see a substantial increase in absorption for photoenergy larger than 2 eV for few-layer SnSe_2_. The layer-dependent excitonic features are revealed in the transmission spectra between 2.0 and 2.2 eV. The absorption of few-layer SnSe_2_ occurs at significantly higher photoenergy as compared to the bulk crystal. This is due to a significant increase in the bandgap of few-layer SnSe_2_ compared to bulk as reported by Oleynik et al. [48]. However, similar to SnS_2_, the bandgap is not sensitive to the thickness for few-layer SnSe_2_ crystals. The red shifts of the excitonic features are due to similar reasons as for SnS_2_. For SnSe_2_, we do not have monolayer and bi-layer samples because they are hard to find under the microscope, probably due to the featureless reflectance contrast in the visible range. In addition, due to the tighter binding of s–p orbital electrons between layers, we found it is harder to isolate few-layer flakes for tin dichalcogenides. For larger atom such as selenium, probably due to the larger affinity, it is more difficult to have mono-layer nanosheet as in the TMDCs.

To the best of our knowledge, this is the first study on layer-dependent optical contrast and spectroscopic features on few-layer SnS_2_ and SnSe_2_. We report the first observation of exciton evolution in few-layer tin dichalcogenides. The tin dichalcogenides exhibit distinct spectroscopic features as compared to the four TMDCs described above. 

## 4. Conclusions

To summarize, we have developed a versatile wide-field hyperspectral microscope, which is capable of capturing high-resolution images in a spectral range of 400 nm to 900 nm. We used the system to efficiently locate few-layer dichalcogenides semiconductor crystal flakes over a centimeter-to-millimeter size area. By using the hyperspectral information to calculate the reflectance contrast and transmission spectra, we can unambiguously determine the number of layers directly during the process of flaking identification. Based on the thickness information of a certain area and the relative contrast between layers, we can facilitate an automatic search of flakes with the desired thickness using the same microscope without having a separate thickness scan. This method has been proven effective for the identification of TMDCs flakes as their excitonic features are prominent in the spectra. We extend this technique to measure SnS_2_ and SnSe_2_, which are indirect semiconductors, regardless of the thickness. We observe distinct spectroscopic features as compared to the TMDCs. Prominent layer-dependent excitonic evolution in transmission spectra is observed. This reveals the effectiveness of our technique for the application in a broader range of materials. Our method is simple, easy to achieve, non-destructive, and reliable. We believe it can not only benefit the fundamental research of 2D materials but also expedite the development process for real-world applications. 

## Figures and Tables

**Table 1:** Comparison of the reflectance contrast peak (*R*_max_), the transmittance minimum (*T*_min_), and the exciton binding energy (*Ex*) of SnS_2_ and SnSe_2_ of different thickness. (^a^ Theoretical exciton binding energy obtained by ab initio calculation [48]; ^b^ Measured exciton binding energy in [53].)

		SnS_2_		SnSe_2_		
Thickness	*R*_max_ (eV)	*T*_min_ (eV)	*Ex* (eV)	*R*_max_ (eV)	*T*_min_ (eV)	*Ex* (eV) ^a^
1-Layer	2.92	1.99	0.91 ^a^	-	-	0.86
2-Layer	2.87	2.19	0.30 ^a^	-	-	0.23
3-Layer	2.83	2.23	0.24 ^a^	2.92	2.14	0.17
4-Layer	2.79	2.26	0.20 ^a^	2.86	2.16	0.14
5-Layer				2.81	2.18	
7-Layer				2.75	2.21	
bulk	2.70	2.23	0.11 ^b^	2.67	2.81	0.093
